# Fatigue in children and adolescents with cancer from the perspective of health professionals**^1^**

**DOI:** 10.1590/1518-8345.1159.2784

**Published:** 2016-08-29

**Authors:** Michele Cristina Miyauti da Silva, Luís Carlos Lopes, Lucila Castanheira Nascimento, Regina Aparecida Garcia de Lima

**Affiliations:** 2 Master's Student, Escola de Enfermagem de Ribeirão Preto, Universidade de São Paulo, PAHO/WHO Collaborating Centre for Nursing Research Development, Ribeirão Preto, SP, Brazil.; 3 Doctoral Student, Escola de Enfermagem de Ribeirão Preto, Universidade de São Paulo, PAHO/WHO Collaborating Centre for Nursing Research Development, Ribeirão Preto, SP, Brazil.; 4 PhD, Associate Professor, Escola de Enfermagem de Ribeirão Preto, Universidade de São Paulo, PAHO/WHO Collaborating Centre for Nursing Research Development, Ribeirão Preto, SP, Brazil.; 5 PhD, Full Professor, Escola de Enfermagem de Ribeirão Preto, Universidade de São Paulo, PAHO/WHO Collaborating Centre for Nursing Research Development, Ribeirão Preto, SP, Brazil.

**Keywords:** Fatigue, Neoplasms, Children, Adolescents, Health Professionals

## Abstract

**Objective:**

to investigate health professionals' knowledge about the concept, assessment and intervention in fatigue in children and adolescents with cancer.

**Method:**

exploratory study with qualitative approach, with 53 health professionals (10 nurses, 33 assistant nurses, 3 physicians, 3 nutritionists, 2 psychologists and 2 physical therapists). Semi structured interviews were held, which were recorded and analyzed by means of inductive thematic content analysis.

**Results:**

the data were organized around three themes: knowledge of health professionals about fatigue; identification of fatigue and interventions to relieve fatigue.

**Conclusion:**

the results indicate the health professionals' limited knowledge about fatigue, as well as the lack of investment in their training and continuing education. Most of all, the lack of research on the theme in the Brazilian context remains a barrier to support improvements in care for this symptom in children and adolescents with cancer.

## Introduction

Cancer-related fatigue (CRF) has been described as the most prevalent symptom in pediatric cancer patients, affecting between 36% and 93% of the cases, with a higher level of fatigue among patients submitted to chemotherapy, affecting between 70% and 100% of the cases^1^ ^-^ ^2^. Children and teenagers consistently report CRF as the most persistent, anguishing, uncomfortable and stressful symptom of cancer and its treatment^1^ ^,^ ^3^. That is why fatigue is a concerning symptom for family members of children and adolescents with cancer as well as health professionals^4^. 

The pathogenesis of CRF has not been fully established yet and a range of mechanisms can contribute to its development. Among these mechanisms, the following stand out: deregulation of pro and anti-inflammatory cytokine levels; activity of the hypothalamic-pituitary-adrenal axis; action of the monoamine system; deregulation of circadian rhythm and alterations in the adenosine triphosphate levels and muscle energy metabolism^5^. Frequent and with variable dimensions, according to the course of the disease, CRF causes a significant negative impact in the functional and psychosocial capacity, reducing the quality of life of cancer patients^3^. Recent studies are reaching a new understanding of subjective symptoms like CRF, stress and quality of life^5^ ^-^ ^7^. 

As aggressiveness and the focus on cure are aspects of the treatment of pediatric oncology, the health team can clinically neglect relevant subjective symptoms like CRF or consider them as unavoidable consequences of the disease and its treatment^8^. Nevertheless, the patients do not always mention fatigue, nor do the health professionals assess or treat this symptom^1^. Hence, often, the family and/or the health team underestimate the CRF, whether due to a lack of understanding of its biological mechanisms, the lack of validated measuring instruments for the child-juvenile population or the patients' hardly clear reports^9^ ^-^ ^10^. Education about fatigue is not always considered a priority though^11^. In addition, the lack of CRF management protocols is a problem that affects care^2^. 

In Brazil, empirical data obtained from a study at a teaching hospital in the State of São Paulo, based on reports of children and adolescents with cancer, revealed their clear discomfort and desire to rest, mainly during the infusion of chemotherapy and the first days afterwards, showing the CRF as a result of the experience of the disease, the chemotherapy or the hospitalization process^12^. 

The importance of knowing the heath professionals' experiences in terms of knowledge, assessment and intervention in CRF in hospitalized children and adolescents with cancer can strongly contribute to specialized care. Besides the clinical and therapeutic assessment, the health professionals need knowledge and sensitivity to perceive the suffering, due to the different reactions that permeate the cancer treatment, mainly focusing on the symptoms^13^. In addition, the multiprofessional health team's control and relief of the CRF are relevant issues that need to be inherent in integrative oncology care^14^. Understanding this phenomenon is a challenge to identify the fatigue as well as to implement measures that can be efficient with a view to achieving a better quality of life for these patients. The objective in this research was to investigate health professionals' knowledge about the concept, assessment and intervention in fatigue in children and adolescents with cancer. 

## Method

An exploratory study with a qualitative approach was undertaken, based on inductive thematic content analysis^15^. The research process took place at the pediatric outpatient clinic and pediatric onco-hematology clinic of a public hospital located in the State of São Paulo, Brazil. Among other specialties, the institution, which was planned for care, teaching and research, is a referral hospital for the treatment of children and teenagers with onco-hematologic disorders.

At the start of the data collection, the multidisciplinary health team was comprised of 72 health professionals, including seven physicians, 12 nurses, 44 assistant nurses, three nutritionists, two psychologists, two physiotherapists and two occupational therapists. Among the health professionals, 53 agreed to participate voluntarily in the study, including 33 assistant nurses, 10 nurses, three nutritionists, three physicians, two psychologists and two physical therapists. In order to guarantee the participants' privacy, we coded each professional category according to their professional background: nurse (NU), nutritionists (N); psychologist (P); physician (PH); assistant nurse (AN) and physical therapist (PT). For example, (NU 4) means [Nurse in interview number 4]. 

The empirical data were collected in 2012, through the application of a semistructured interview with a mean duration of 45 minutes. The interviews were digitally recorded and then fully transcribed. A script was elaborated to guide the interview, which consisted of two parts: the first addressed the participants' sociodemographic characteristics, such as age; sex; marital status; with or without child, educational level, profession; time since graduation; length of experience in the team and continuing education activities; and the second part contained the following guiding questions: *a) What is fatigue?; b) How do you identify and assess a child or teenager with cancer with fatigue?* and *c) What interventions are used to relieve the fatigue?*

The empirical data were analyzed by means of inductive thematic content analysis, as this flexible method grants theoretical freedom and respect for the coherence between the research problem and the objective. It consists of six phases *a)* approach of the data; *b)* identification and coding of the data; *c)* revision of coded units (manually or using software) and construction of the themes; *d)* revision and refining of the themes; *e)* naming of the thematic axes and identification of the essence of the themes and *f)* signification/interpretation of the emerging themes^15^. It is highlighted that the inductive thematic modality permits the extraction of themes from the data, as a data coding process that does not drift on the preexisting theoretical structure.

The second analysis phase was developed manually, when each unit of meaning was marked with distinct colors. Therefore, the empirical material was subject to exhaustive readings, in the attempt to identify words, excerpts and semantic similarities repeated in each interview, followed by their indexation and content analysis. In excerpts from testimonies in which the word fatigue corresponded to its definition, for example, the color green was used; when it referred to interventions and/or practical actions for its management, the color blue was used, and so forth. In this phase, looking for codes of meanings and their hierarchies, various pre-themes emerged, which then constituted 11 thematic units. These were cautiously reanalyzed to exclude subthemes that were repeated to better represent the empirical material and its convergence with the research objective, which was a careful process during the analysis.

This study followed the recommendations of National Health Council Resolution 196/1996 in force at the time, in compliance with the ethical requirements for research involving human beings. The project received approval from the Institutional Review Board at the University of São Paulo at Ribeirão Preto Medical School under protocol number 1391/2011. In addition, in the attempt to protect the research participants' integrity, authorization was obtained to record the interview with the help of a digital recording, as well as authorization from each technical director of the professional entity to interview the participants during the work hours.

## Results

Fifty-three health professionals participated in the study. The respondents' sociodemographic characteristics are displayed in Table 1. The empirical material in this research was organized around three themes: knowledge about fatigue, presented according to the professionals' experience on this symptom; identification of fatigue, picturing how the interviewees perceived it in the children and adolescents with cancer and intervention to relieve the fatigue, presented based on the interviewees' actions to manage it. 

**Table 1:** Socio-demographic characteristics of participants. Ribeirão Preto, SP, Brazil. 2014.

Variable		n	%
Sex			
	Female	48	90,6
	Male	5	9.4
Age group			
	20-30	13	24.6
	31-40	16	30.2
	41-50	14	26.4
	51-60	10	18.9
Marital status			
	Single	16	30.2
	Married	33	62.3
	Divorced	3	5.7
	Widow	1	1.9
With children			
	Yes	34	64.2
	No	19	35.8
Education level			
	Technical	27	50.9
	Higher education	13	24.5
	Postgraduate	13	24.5
Formation time (years)			
	0-10	20	37.8
	11-20	21	39.6
	21-30	11	20.7
Permanent education			
	Yes	5	9.4
	No	48	90.6
Qualifications			
	Auxiliary nurse	33	62.2
	Nurse	10	18.9
	Physician	3	5.6
	Nutritionist	3	5.6
	Physical therapist	2	3.8
	Psychologist	2	3.8
Years of work			
	0-10	25	47.1
	11-20	20	37.7
	Above 20	8	15.1

### Knowledge about fatigue

As to the knowledge about cancer-related fatigue, the professionals use their clinical experiences to explain this concept: *... We perceive it based on our experience. Some do express it, some tell it ...* (AN 27).

 ... *When I think of fatigue, I think of tiredness ...* (NU 4).

Some testimonies revealed that there was no consensus on the fatigue concept and that, for some, this concept was not based on the scientific literature. Fatigue was understood as it is experienced in clinical practice, expressing that its concept and mechanisms were not clear yet for the multidisciplinary team members, even for those who had worked in pediatrics for several years.

A definition the participants frequently expressed refers to fatigue as tiredness: *... It's her discomfort. Tired! She is physically tired ... you perceive the child's fatigue and disquiet...* (NU 3).

... *For me, fatigue in children with cancer is abnormal tiredness, feels weakness ... and this fatigue even comes from him not being active. When he's resting he feels tired. He is unable to sleep adequately. No matter how much he sleeps, he wakes up complaining of tiredness, of weakness, of feeling bad ...* (NU 5). 

... *I think that what comes to my mind with regard to fatigue is a more chronic tiredness, a continuing tiredness ...* (P 2).

For some participants, the knowledge about fatigue is translated as a stressful situation a child or teenager with cancer experiences: *... Fatigue I associate with matters of stress...* (P 1).

*... It's stress. The child gets stressed, irritated, then you notice that she does not even want you ... When she'll be hospitalized and when she'll start the treatment ...* (AN 1).

... *Fatigue, I think it's the tiredness of coping with disease (stress), of expecting cure, improvement and getting tired and not wanting anymore, losing the desire ...* (PH 2).

*... The child cannot distinguish that, but I believe that it's great stress she may be going through, but without her knowing it. It's not tiredness, neither physical nor mental, but it can be considered as a stressful period; so I don't know how the child can reflect that... I think she encompasses it all, it may even lead to a depression ...* (NU 1).

Some participants mentioned doubts and even lack of knowledge of the symptom: *... Fatigue, I think it's despair, I don't know. How can I explain? ... An anxiety ... a fear ... stress ... fatigue ends up being a mixture of a lot of emotions? I don't know ...* (AN 33).

### Identification of fatigue

In the testimonies, it was observed that the fatigue symptom has been recognized based on the children or teenagers' expressions: *... The child tells you: "look, I'm feeling bad, I'm out of breath, I'm so tired" ...* (NU 2).

*... Some express a bad mood, stress, or when the patient is being indelicate to you...* (PH 1).

*... I'm so tired, I'm sad, I can't take it anymore! They say it, it's characteristic of the child. When they are feeling well, they come and tell you: "I'm fine"... when they're not well, they say: "I'm not well" ...* (N 1).

*... First I listen to the child's report, her statement. The report of the mother or relatives or a caregiver, who talks about some symptoms to characterize the fatigue and the children's report. The children who are able to use speech, expression, further, they do so. The issue of pain is also closely associated ...* (P 1).

According to the professionals, the fatigue is strongly related to the patients' emotional and psychological dimension, as: *... When the child starts to appear very discouraged, very apathetic, she gets very isolated, hardly active, you notice that there is a cognitive debasement, lack of stimulation ... She does not want to play, does not want to talk to other people. She appears discouraged, sleepy, with a lack of appetite, which causes difficulties in daily life, in relationships, entails isolation, depressive symptoms, so I think that's more or less it ...* (P 1).

On the other hand, lack of knowledge about the identification of a fatigue situation is identified: *... I cannot identify it, to the extent that I said that many professionals do not know it, they cannot even identify it ...* (AN 8).

### Fatigue relief interventions

One participant focused her response on a respiratory fatigue situation, emphasizing the nursing care: *... First, I try to see if she's receiving oxygen or not. If she's not, I install the support. Then, I look for comfort measures, like raising the headrest, a simple measure, but which improves a lot; so I raise the headrest. Sometimes raising limbs to improve the oxygenation as well. If it's a child whom I notice is unable to purify the secretion I aspire. Some nursing care I assess at that moment and do it. Sometimes I talk to the physician to check if there's a need for medication to improve it. It's more analgesia to try and improve the feeling of pain there a little ...* (NU 2).

According to the participant, this care can involve: position change in the bed, raising limbs, aspiration of secretion, massage, communication to the physician of the possible need for medication, among other interventions. 

The intervention one physician mentions refers to the relation physician-patient and physician-multidisciplinary team. This professional highlighted that, often, the child was in doubt about her disease and treatment and, thus, he tried to talk to her and to the team: *... Talk! Sometimes this patient was unable to talk, sometimes technical information was transmitted, but sometimes they're very quick. So, an intervention is needed, to stop, talk and explain, mainly explain the purpose of these procedures. We have a joint meeting where we discuss the patients hospitalized that week, the different viewpoints of psychology, nutrition, social service, the cases that need to be further discussed* (PH 1).

One of the psychologists highlighted that efforts are needed to include the child in socializing activities, as the need to stay in hospital for a long time prevents the children from attending school and having contact with other children of their age. The professional also emphasized that the pain the invasive procedures the child is submitted to cause can lead to depression: *... A motivation, in socialization as well, because I think that a child is able to interact well. I think it's a positive sign, an important point against apathy and in favor of playing! It encourages a lot. We notice that a child is well through playing. When you interact with her, offer the playful and the child refuses, she gets very apathetic, there's no interaction. I find it very important to have a psychologist, an occupational therapist to work with the playful, providing activities to entertain the child, to help with coping* (P 1).

The same professional mentioned that these interventions can demand short monitoring by a physical therapist and, when necessary, psychiatric assessment and even medication: *... Intervention through psychotherapy we do here at the hospital, a short psychotherapy, focused, to help and cope with the hospitalization. Of course if the child and mother mention that this (difficulties) continues at home, we will work in another way, forwarding, sometimes requesting a psychiatric assessment, I feel that it's all related, the fatigue issue, the depressive symptoms ...* (P 1).

One assistant nurse mentioned some intervention actions, including: talks with the child, telling stories, songs and games: *... I talk, I tell a story, I sing, I play, it gets better ...* (AN 1).

As observed, these are activities related to the daily life of a healthy child that, in the hospital context, are intended to offer close-to-normal conditions.

## Discussion

The discussion of this research is focused on the process of care as well as the clinical competence for the implementation of this care. When an individual is questioned about a certain concept, he can answer based on his professional and personal experience. To elaborate a concept, theoretical and practical knowledge is needed to interpret and subjectively deal with each professional's experience, because the concept is a thought, a notion or a particularly important idea for the construction of knowledge^16^. 

The association between fatigue and tiredness was present in the interviewees' testimonies and its identification was mainly based on the reduction of the functional capacity or the capacity to accomplish daily activities, lack of energy, reduced motivation, aversion to activities, suffering and extreme need for rest. These testimonies are in line with different authors' concepts, who also describe fatigue as abnormal tiredness that does not stop with rest periods^8^ ^-^ ^9^. In a Brazilian study aimed at analyzing this concept, it was demonstrated that the manifestation of tiredness or exhaustion, negatively affecting the development of daily activities, and the impossibility to recover energy through common strategies are identified as the main empirical references of the fatigue concept for the health area^9^. 

The testimonies regarding the fatigue concept indicate that different health professionals relate fatigue to stress - physical stress or deriving from a long period of hospitalization and treatment, or caused by the uncertainty of the disease prognosis. This association is probably due to the fact that fatigue is frequently associated with psychological factors, the course of the treatment and the disease phase^2^ ^,^ ^17^. Different psychophysiological explanatory models of CRF have been proposed, many of which use similar constructs^5^ ^-^ ^7^ ^,^ ^17^. For instance, the Fatigue Adaptation Model (FAM) presents a framework for the study of fatigue, which refers to a possible link between behavioral and physiological indices of tiredness, fatigue, and exhaustion as they occur in both ill and non-ill states. The central thesis of this approach is that stressors related to disease processes in cancer and to its treatment, declines cognition, sleep quality, nutrition and muscle endurance. This decline inhibits the ability to adapt to stressors increasing the risk to develop fatigue. Due to this fact, fatigue is considered a stress response and defined as a marker for the inability to adapt to stressors caused by the disease and treatment. In that framework, it is observed that tiredness and exhaustion constitute a continuum of adaptive response to that situation, which can come with different behavior characteristics and symptoms^7^.

Doubts regarding prevalence, identification and management result in mistakes in the understanding of the symptom and, consequently, in the moment when effective interventions are proposed^9^. A study showed that the nurses define fatigue mainly as tiredness (62%), lethargy (45%), weakness (32%), psychological effects (32%) and lack of energy (24%)^16^. This lack of agreement and consensus is also reflected in the health professionals' clinical practice, as the study participants revealed. Knowing the particularities of each symptom, particularly of fatigue, is fundamental to improve the intervention strategies and to advance in the development of knowledge to manage this symptom. 

As a fundamentally subjective condition, the patients can experience and express fatigue in distinct forms^8^ ^,^ ^18^. According to the nursing professionals, for example, the orientation to recognize and intervene in patients in fatigue situations follows the recommendations of the North American Nursing Diagnosis Association. These contain, as characteristics, verbal and behavioral signs and symptoms. The former - verbalization of a constant and oppressive lack of energy and increase of physical and behavioral complaints - cognitive debasement, disinterest in the surrounding environment, incapacity to maintain the common level of physical activity, introspection, lethargy, sleepiness and feeling of guilt for not complying with the responsibilities^19^. 

The nurses need to accept fatigue as a concrete and relevant symptom of the cancer treatment and be aware of its manifestation. One important step is the incorporation of fatigue in the routine nursing assessments of children and adolescents with cancer. The acute recognition and identification of physical or mental fatigue indicators, such as mood swings, reduced communication or isolation, are fundamental for the early diagnosis. Mood, distraction, diet, self-monitoring of hematologic and immunologic systems, making the hospital environment less noisy and decreasing the sleep interruptions, are efficient interventions to manage this symptom, which these professionals can conduct^20^.

A study undertaken in Turkey in 2010 was aimed at describing how the health professionals define and assess fatigue in children with cancer and indicated the following signs to identify the fatigue: lethargy (78.6 %, n = 44), reduced appetite (39.3 %, n = 22), mood swings (39.3%, n = 22), tiredness (35.7%, n = 20), sleep alterations (32.1%, n = 18), disinterest (28.6 %, n = 16), insomnia (38.3%, n = 18) and incapacity to accomplish daily activities (25.0%, n = 8)^21^. In this sense, these data converge with some of the clinical characteristics most of the health professionals in this research report, who associate it mainly with tiredness, lethargy, mood swings and disinterest.

A study undertaken in Greece to assess the change in fatigue scores during the cancer treatment according to 40 children, 29 teenagers and their parents and to describe the possible causes of this symptom demonstrated that children (F = 6.85, p = 0.00), adolescents (F = 4.15, p = 0.03) and their parents (F = 3.98, p = 0.02) showed a statistically significant increase in fatigue scores during the treatment. The hospital environment and the medical procedures were assessed as the factors that most contributed to the treatment-related fatigue in the three groups^20^. 

Children and teenagers under treatment for cancer can experience different psychoneurological symptom clusters, which frequently include fatigue. These symptom groups normally take place simultaneously, and mainly involve pain, nausea, sleep disorders, anguish, suffering and fear. It is believed that this can be one of the aspects that hamper the diagnosis of the fatigue. It is highlighted that the psychoneurological symptom clusters are associated with worse prognoses, low survival, behavioral changes and psychophysiological performance disorders, reduced treatment adherence and lower health-related quality of life^13^ ^-^ ^14^. 

 Difficulties to recognize and manage fatigue include the fact that it is a subjective symptom, which is not life threatening and is considered an unavoidable consequence by the health team. In addition, in most cases, fatigue is underdimensioned and, therefore, the health professionals may not fully perceive the level of suffering and functional loss it causes^4^. In children and adolescents, however, tools have been used to assess and identify fatigue^22^ ^-^ ^24^, including: Childhood Fatigue Scale (CF-S); Parent Fatigue Scale (PF-S); Staff Fatigue Scale (SF-S)*,* all of which were developed by the same author^25^ and the PedsQL Multidimensional Fatigue Scale^10^. 

A cross-sectional study, conducted in 11 European countries, using a paired sample of 1,933 patients and health professionals to investigate the level of agreement between the patients and health professionals' assessments of cancer symptoms, indicated that 71% of the patients assessed the fatigue symptom as moderate and that 54% of the professionals assessed it as severe. The health professionals underestimated the symptoms in approximately one out of ten patients^11^.

Another study revealed that the patients often hesitate to spontaneously mention the fatigue to their physicians because these professionals associate it with the natural progression of the disease and, in the desire to be "good patients", they end up ignoring the pharmacological and non-pharmacological treatment options to manage this symptom. In addition, CRF patients mention a feeling that the health professionals do not recognize the tiredness^26^. Most physicians are unable to offer effective interventions to manage this symptom, due to the lack of information about fatigue and education for health professionals, patients and their families^27^ ^-^ ^28^.

The professionals need technical-scientific qualification to identify the symptom, which can be achieved through continuing education programs. This is a route towards the emancipation and autonomy of health professionals, as it is at the crossing between professional education and the job world that learning and teaching are incorporated into the institutions' daily reality^29^. 

Managing CRF represents a great challenge for the health professionals, but the interventions in children and teenagers with cancer that are being developed or tested to managed this symptom are still insufficient, mainly due to the methodological quality of the clinical trials conducted, sample size problems and difficulties to recruit participants because of the parents' overprotection^14^ ^,^ ^27^. 

The treatment success depends on an approach mediated by health education, which considers the nature of the fatigue, the therapeutic complementarities and the possible problems. Due to the distinct causes of fatigue, the therapeutic plan should be multidimensional and individual, also recommending the family's engagement for the sake of a better assessment of the symptom and identification of management strategies^4^. 

Although fatigue is often mainly described as a physical symptom in the literature, promoting interventions that only consider this dimension and its biological manifestations, this symptom interferes in several areas of the patients' lives, affecting physical, cognitive, affective and behavioral domains and can be experienced at different levels, ranging from mild to severe^4^ ^,^ ^7^ ^,^ ^28^. The psychological and social support the patients require can vary depending on their fatigue level and interventions that strengthen the social support network, protecting them against the disease-related stress^27^ ^-^ ^28^. In addition, as part of a comprehensive assessment, it is important to closely listen to the patient's fatigue experience in order to provide information on the possible underlying cause^4^. 

Although people with cancer commonly mention that they are tired or feel "shattered" or "out of energy", the assessment of fatigue often is not routine practice. Nevertheless, it is important to closely listen to the patients and place their observations in a context, as part of a comprehensive assessment. Treatable causes like anemia, infections, malnutrition, dehydration and depression need to be taken into account and treated. As the patients' physical, cognitive and mental dimensions are frequently interlinked though, it may not be possible to distinguish between a downhearted mood caused by an underlying depression or by a physical lack of energy. A precise assessment is needed in order to identify the actual problem^4^ ^-^ ^5^. 

As a non-pharmacological intervention strategy to relieve the symptom, the literature recommends activities that permit distraction, social life, work and even the encouragement to solve problems in order to deviate the patient's focus from the fatigue situation^13^. General care recommendations to support the patients in the attempt to enhance the CRF treatment include the observation of the nutritional status and the prevention of weight loss, balancing rest with assisted physical exercise and mainly including measures that permit restoration and distraction. Examples are music therapy and dramatic therapeutic play^2^ ^-^ ^3^ ^,^ ^13^. 

A Turkish study about fatigue assessment in children with cancer revealed that the interventions used to reduce the symptom were: talking to the patient about the symptom to provide information and nutritional support, besides promoting relaxation activities^21^. A recent systematic review and meta-analysis that synthesized scientific evidence available between 1960 and 2010 about the effect of non-pharmacological interventions to control fatigue in children and teenagers with cancer identified six studies, four of which involved programmed physical exercise, one therapeutic massage and one nursing interventions to relieve CRF. Among the studies, statistical significance was verified for interventions that used physical exercise to reduce fatigue in that population (effect magnitude = -0.76; CI_95%_= [-1.35, -0.17])^2^. 

The increased knowledge on fatigue has not sufficiently encouraged the patients to discuss the symptom with their physicians and nurses. The patients cope with the symptom intuitively, as well as the health team, despite valid and reliable assessment instruments and a range of effective management instruments^1^ ^,^ ^4^. The health professionals hardly use these guidelines and the patients do not know which interventions can relieve the fatigue. The oncologists and oncologic nurses who take care of these patients are challenged to implement the available scientific evidence in their practice^4^. 

A limitation of the study is the analysis of data performed globally, i.e. with the healthcare team interchangeably. Thereby, this option did not consider the singularity of the different professional categories separately. In addition, other studies should address the particularities of the knowledge of physicians, nurses, psychologists, occupational therapists and nutritionists on fatigue in children and adolescents with cancer in order to contribute to the management of this symptom from the multidisciplinary approach.

## Conclusion

These research findings permitted describing which knowledge that health professionals have about the concept, assessment and intervention on fatigue in children and adolescents with cancer. Based on the results, the knowledge about CRF is not clear for most of the health professionals, as they mainly rest on their professional experience. In addition, some difficulty was found among the professionals in terms of the identification of CRF. Most of the interviewees showed confusion between the concomitant symptoms the children and teenagers with cancer present and the etiology of fatigue. Concerning the interventions these professionals accomplish to manage and relieve the fatigue, these remain restricted to some limited actions, while other recommended interventions with available clinical evidence are unknown. These findings indicate the investigated health professionals' limited knowledge on different aspects of the management of cancer-related fatigue, and also evidence the lack of training to take care of these patients.

 In addition, the lack of studies on CRF in children and adolescents, especially in the Brazilian literature, from the multiprofessional perspective, minimizes the possibilities of developing strategies for its recognition, hampering the systemization of successful therapeutics. Thus, this research contributed to support the development of effective strategies, as it alerted to the clinical relevance of the cancer-related fatigue symptom, highlighting the fundamental role of health professionals in the multidisciplinary construction of the management of this symptom.
